# Supplementary material for: Single Cell Transcriptomic and Chromatin Profiles Suggest Layer Vb Is the Only Layer With Shared Excitatory Cell Types in the Medial and Lateral Entorhinal Cortex
Source: Front Neural Circuits. 2022 Jan 26;15:806154. doi: 10.3389/fncir.2021.806154 (PMC8826650; doi:10.3389/fncir.2021.806154)
Supplement: Supplementary file 14 [file Data_Sheet_1.ZIP › 11/knownResults.html]

11 - Homer Known Motif Enrichment Results


# Homer Known Motif Enrichment Results (11)

Homer *de novo* Motif Results  
Gene Ontology Enrichment Results  
Known Motif Enrichment Results (txt file)  
Total Target Sequences = 36093, Total Background Sequences = 35491

|  |  |  |  |  |  |  |  |  |  |  |  |
| --- | --- | --- | --- | --- | --- | --- | --- | --- | --- | --- | --- |
| Rank | Motif | Name | P-value | log P-pvalue | q-value (Benjamini) | # Target Sequences with Motif | % of Targets Sequences with Motif | # Background Sequences with Motif | % of Background Sequences with Motif | Motif File | SVG |
| 1 | A G C T A C G T A C T G A T G C A G T C C G T A C T G A T A C G | NF1-halfsite(CTF)/LNCaP-NF1-ChIP-Seq(Unpublished)/Homer | 1e-615 | -1.417e+03 | 0.0000 | 14199.0 | 39.34% | 9398.0 | 26.48% | motif file (matrix) | svg |
| 2 | T G C A A G C T C T G A A T C G G A C T C T A G G T A C G A T C G T C A A G T C G T A C G A C T C T A G A T C G G C A T C A T G C A T G G A T C G T A C C T G A | CTCF(Zf)/CD4+-CTCF-ChIP-Seq(Barski\_et\_al.)/Homer | 1e-590 | -1.359e+03 | 0.0000 | 2523.0 | 6.99% | 729.7 | 2.06% | motif file (matrix) | svg |
| 3 | A G T C G A C T C A G T A C T G C T A G T G A C G C T A A T G C G C A T A T C G C G A T A C T G G A T C G T A C G T C A C T G A | NF1(CTF)/LNCAP-NF1-ChIP-Seq(Unpublished)/Homer | 1e-571 | -1.316e+03 | 0.0000 | 4526.0 | 12.54% | 1934.1 | 5.45% | motif file (matrix) | svg |
| 4 | A G C T G T C A C G T A A C G T A C G T C T G A T C A G A T G C | Lhx2(Homeobox)/HFSC-Lhx2-ChIP-Seq(GSE48068)/Homer | 1e-422 | -9.720e+02 | 0.0000 | 7442.0 | 20.62% | 4394.2 | 12.38% | motif file (matrix) | svg |
| 5 | A T G C T A C G A G T C G A C T T G C A C T G A G A C T A C G T C T G A T C A G | LHX9(Homeobox)/Hct116-LHX9.V5-ChIP-Seq(GSE116822)/Homer | 1e-384 | -8.864e+02 | 0.0000 | 9387.0 | 26.01% | 6099.7 | 17.18% | motif file (matrix) | svg |
| 6 | T C A G C T A G A T G C G A C T T G C A C G T A A G C T A C G T C T G A T A C G | En1(Homeobox)/SUM149-EN1-ChIP-Seq(GSE120957)/Homer | 1e-360 | -8.293e+02 | 0.0000 | 11388.0 | 31.55% | 7908.0 | 22.28% | motif file (matrix) | svg |
| 7 | C G T A C A T G A G T C G A C T T G C A C G T A A C G T A C G T C T G A T C A G | Lhx1(Homeobox)/EmbryoCarcinoma-Lhx1-ChIP-Seq(GSE70957)/Homer | 1e-352 | -8.123e+02 | 0.0000 | 7626.0 | 21.13% | 4762.7 | 13.42% | motif file (matrix) | svg |
| 8 | T A G C A G T C T G A C A G T C C T A G A T C G A G T C C A T G T G A C A G T C G T A C A G T C A G T C G C A T C T A G A T C G G C A T A C T G A T C G G A T C | BORIS(Zf)/K562-CTCFL-ChIP-Seq(GSE32465)/Homer | 1e-300 | -6.930e+02 | 0.0000 | 2980.0 | 8.26% | 1393.5 | 3.93% | motif file (matrix) | svg |
| 9 | A T C G A T C G A T G C G A C T T C G A C G T A G C A T A G C T C T G A T A C G | DLX2(Homeobox)/BasalGanglia-Dlx2-ChIP-seq(GSE124936)/Homer | 1e-300 | -6.921e+02 | 0.0000 | 9283.0 | 25.72% | 6342.8 | 17.87% | motif file (matrix) | svg |
| 10 | C G T A C G T A A G C T G A C T T G C A G T C A A C G T A G C T C T G A T C A G | Lhx3(Homeobox)/Neuron-Lhx3-ChIP-Seq(GSE31456)/Homer | 1e-298 | -6.872e+02 | 0.0000 | 10095.0 | 27.97% | 7050.8 | 19.86% | motif file (matrix) | svg |
| 11 | A G T C C G A T A C T G A T C G T G A C G C T A C A T G A T C G T G A C C G A T A C T G T A G C G T A C G T C A | Tlx?(NR)/NPC-H3K4me1-ChIP-Seq(GSE16256)/Homer | 1e-285 | -6.562e+02 | 0.0000 | 4184.0 | 11.59% | 2286.7 | 6.44% | motif file (matrix) | svg |
| 12 | A T G C T A C G A G C T T G C A C G T A C G A T A C G T C T G A | DLX5(Homeobox)/BasalGanglia-Dlx5-ChIP-seq(GSE124936)/Homer | 1e-278 | -6.408e+02 | 0.0000 | 5339.0 | 14.79% | 3186.6 | 8.98% | motif file (matrix) | svg |
| 13 | T C G A C A G T A C T G A G C T C G T A C G T A A C G T A C G T C T G A T A G C | Dlx3(Homeobox)/Kerainocytes-Dlx3-ChIP-Seq(GSE89884)/Homer | 1e-273 | -6.299e+02 | 0.0000 | 4477.0 | 12.40% | 2537.9 | 7.15% | motif file (matrix) | svg |
| 14 | A C T G C A G T A C G T C G T A C G T A A C G T A C T G C T G A | Nkx6.1(Homeobox)/Islet-Nkx6.1-ChIP-Seq(GSE40975)/Homer | 1e-241 | -5.558e+02 | 0.0000 | 13523.0 | 37.47% | 10408.9 | 29.32% | motif file (matrix) | svg |
| 15 | A T C G A T G C A G T C T A G C G A C T T C G A G C T A G C A T A G C T C T G A | DLX1(Homeobox)/BasalGanglia-Dlx1-ChIP-seq(GSE124936)/Homer | 1e-233 | -5.385e+02 | 0.0000 | 8124.0 | 22.51% | 5645.2 | 15.90% | motif file (matrix) | svg |
| 16 | A G T C C G A T C T G A C G T A A C G T C A G T T C A G T G A C | Isl1(Homeobox)/Neuron-Isl1-ChIP-Seq(GSE31456)/Homer | 1e-180 | -4.150e+02 | 0.0000 | 11164.0 | 30.93% | 8619.1 | 24.28% | motif file (matrix) | svg |
| 17 | G C T A C G T A A G T C A C G T T C G A T A C G A C T G A G C T A G T C T C G A | RORgt(NR)/EL4-RORgt.Flag-ChIP-Seq(GSE56019)/Homer | 1e-174 | -4.018e+02 | 0.0000 | 1279.0 | 3.54% | 513.5 | 1.45% | motif file (matrix) | svg |
| 18 | G C T A C G T A A G T C A C G T T C G A T A C G A C T G A G C T A G T C T C G A | RORgt(NR)/EL4-RORgt.Flag-ChIP-Seq(GSE56019)/Homer | 1e-174 | -4.018e+02 | 0.0000 | 1279.0 | 3.54% | 513.5 | 1.45% | motif file (matrix) | svg |
| 19 | C G T A C G T A C G T A A G T C A G C T C T G A A C T G C T A G A G C T A G T C C G T A T C A G | RORg(NR)/Liver-Rorc-ChIP-Seq(GSE101115)/Homer | 1e-165 | -3.822e+02 | 0.0000 | 963.0 | 2.67% | 338.1 | 0.95% | motif file (matrix) | svg |
| 20 | G A C T C T A G A T G C A G T C G T C A T A C G A T G C A T C G | HIC1(Zf)/Treg-ZBTB29-ChIP-Seq(GSE99889)/Homer | 1e-158 | -3.659e+02 | 0.0000 | 14341.0 | 39.74% | 11705.5 | 32.98% | motif file (matrix) | svg |
| 21 | C G T A C G T A C G T A A G T C A G C T C T G A A C T G A C T G A G C T A G T C C G T A C T A G C T A G C T A G T G C A | RORa(NR)/Liver-Rora-ChIP-Seq(GSE101115)/Homer | 1e-140 | -3.239e+02 | 0.0000 | 1296.0 | 3.59% | 581.8 | 1.64% | motif file (matrix) | svg |
| 22 | C T A G A G C T G A C T C A T G A G T C A G T C G T C A C A G T C T A G T C A G G T A C C T G A T C G A G A T C T G A C | Rfx2(HTH)/LoVo-RFX2-ChIP-Seq(GSE49402)/Homer | 1e-119 | -2.745e+02 | 0.0000 | 1013.0 | 2.81% | 437.4 | 1.23% | motif file (matrix) | svg |
| 23 | C A G T T C A G A G C T G A C T A C G T A G T C G A T C G A C T C T G A A C T G G A T C C G T A C T G A A G T C G T A C | Rfx6(HTH)/Min6b1-Rfx6.HA-ChIP-Seq(GSE62844)/Homer | 1e-112 | -2.586e+02 | 0.0000 | 7565.0 | 20.96% | 5825.4 | 16.41% | motif file (matrix) | svg |
| 24 | C G T A C T A G T C A G T C A G A G T C A T G C A G T C G C A T A G C T A C G T A T C G C G A T | Sox9(HMG)/Limb-SOX9-ChIP-Seq(GSE73225)/Homer | 1e-111 | -2.564e+02 | 0.0000 | 5856.0 | 16.23% | 4326.0 | 12.19% | motif file (matrix) | svg |
| 25 | A G T C A T C G C T A G A G C T G A C T C T A G A G T C A G T C G C T A C A G T T C A G T C A G G A T C C T G A T C G A G A T C | RFX(HTH)/K562-RFX3-ChIP-Seq(SRA012198)/Homer | 1e-101 | -2.342e+02 | 0.0000 | 933.0 | 2.59% | 417.8 | 1.18% | motif file (matrix) | svg |
| 26 | A T G C A G T C G C A T A G C T A C G T T C A G C G A T A G C T G A T C A T C G | Sox10(HMG)/SciaticNerve-Sox3-ChIP-Seq(GSE35132)/Homer | 1e-97 | -2.249e+02 | 0.0000 | 9859.0 | 27.32% | 8016.8 | 22.59% | motif file (matrix) | svg |
| 27 | A T G C G A T C C G A T A C G T A C G T A C T G C A G T A G C T | Sox3(HMG)/NPC-Sox3-ChIP-Seq(GSE33059)/Homer | 1e-90 | -2.089e+02 | 0.0000 | 10247.0 | 28.39% | 8434.4 | 23.76% | motif file (matrix) | svg |
| 28 | C A T G C T A G A G C T G A C T C A T G A G T C G A T C G C T A C G A T C T A G T C A G G T A C C T G A T C G A | X-box(HTH)/NPC-H3K4me1-ChIP-Seq(GSE16256)/Homer | 1e-85 | -1.978e+02 | 0.0000 | 1046.0 | 2.90% | 526.1 | 1.48% | motif file (matrix) | svg |
| 29 | T G A C C T G A C T A G C T G A C G T A A G T C C T G A A C G T G C A T T A G C G C A T A T C G G A C T G A C T G A T C | GRE(NR),IR3/RAW264.7-GRE-ChIP-Seq(Unpublished)/Homer | 1e-81 | -1.888e+02 | 0.0000 | 1581.0 | 4.38% | 927.1 | 2.61% | motif file (matrix) | svg |
| 30 | A C T G T C A G A G C T G A C T C A T G A G T C A G T C G C T A C G A T C T A G T C A G G T A C C T G A T C G A | Rfx1(HTH)/NPC-H3K4me1-ChIP-Seq(GSE16256)/Homer | 1e-78 | -1.807e+02 | 0.0000 | 1748.0 | 4.84% | 1067.8 | 3.01% | motif file (matrix) | svg |
| 31 | C G T A C T G A C T A G C G T A C G T A A G T C C G T A C A G T G C A T G T C A C G A T A C T G A C G T G C A T G A T C | PGR(NR)/EndoStromal-PGR-ChIP-Seq(GSE69539)/Homer | 1e-77 | -1.790e+02 | 0.0000 | 1421.0 | 3.94% | 820.1 | 2.31% | motif file (matrix) | svg |
| 32 | A G T C A G T C C G A T A C G T A C G T A C T G A C G T A G C T A G T C A G T C | Sox4(HMG)/proB-Sox4-ChIP-Seq(GSE50066)/Homer | 1e-70 | -1.629e+02 | 0.0000 | 5481.0 | 15.19% | 4268.6 | 12.03% | motif file (matrix) | svg |
| 33 | G A C T C G T A A C G T A C T G A G T C C G T A C T G A C G T A C A G T A C G T G T C A T C A G | Brn1(POU,Homeobox)/NPC-Brn1-ChIP-Seq(GSE35496)/Homer | 1e-69 | -1.611e+02 | 0.0000 | 1885.0 | 5.22% | 1207.3 | 3.40% | motif file (matrix) | svg |
| 34 | G C A T C G T A C G A T A C T G A G T C G C T A C T G A C G T A C A G T A C T G C G T A T C A G | Oct6(POU,Homeobox)/NPC-Pou3f1-ChIP-Seq(GSE35496)/Homer | 1e-67 | -1.553e+02 | 0.0000 | 2462.0 | 6.82% | 1686.2 | 4.75% | motif file (matrix) | svg |
| 35 | T A C G C T G A C T A G T C G A C G T A A G T C C T G A A T C G G C A T T A G C G A C T A C T G A C G T A G C T A G T C G A C T | GRE(NR),IR3/A549-GR-ChIP-Seq(GSE32465)/Homer | 1e-66 | -1.540e+02 | 0.0000 | 990.0 | 2.74% | 534.1 | 1.50% | motif file (matrix) | svg |
| 36 | C T G A T C G A C G T A A T G C C G T A C G T A C G A T C T A G T C A G G A T C | Sox15(HMG)/CPA-Sox15-ChIP-Seq(GSE62909)/Homer | 1e-66 | -1.537e+02 | 0.0000 | 6321.0 | 17.51% | 5052.5 | 14.23% | motif file (matrix) | svg |
| 37 | A T G C A G T C G A T C C G T A A C G T A C G T A C T G A C G T A G C T G A T C | Sox2(HMG)/mES-Sox2-ChIP-Seq(GSE11431)/Homer | 1e-64 | -1.492e+02 | 0.0000 | 5471.0 | 15.16% | 4305.1 | 12.13% | motif file (matrix) | svg |
| 38 | A T G C G T A C C G T A A G C T G C A T T A C G A G C T A G C T A G T C A G C T | Sox6(HMG)/Myotubes-Sox6-ChIP-Seq(GSE32627)/Homer | 1e-64 | -1.477e+02 | 0.0000 | 8972.0 | 24.86% | 7503.3 | 21.14% | motif file (matrix) | svg |
| 39 | C T A G T A C G G A T C G T A C G C T A A G C T A G C T G T C A T C G A T A G C | Nanog(Homeobox)/mES-Nanog-ChIP-Seq(GSE11724)/Homer | 1e-64 | -1.475e+02 | 0.0000 | 20629.0 | 57.16% | 18710.1 | 52.71% | motif file (matrix) | svg |
| 40 | G C T A G C A T G A C T G C A T T C A G G T A C G C T A G C A T C T G A G C T A T A G C G C T A C T G A C G A T C T A G | OCT4-SOX2-TCF-NANOG(POU,Homeobox,HMG)/mES-Oct4-ChIP-Seq(GSE11431)/Homer | 1e-58 | -1.343e+02 | 0.0000 | 1167.0 | 3.23% | 691.6 | 1.95% | motif file (matrix) | svg |
| 41 | T C A G A G C T A C G T A C G T G T A C G A T C C G T A C T A G C A T G G T C A C G T A T C G A | STAT4(Stat)/CD4-Stat4-ChIP-Seq(GSE22104)/Homer | 1e-52 | -1.216e+02 | 0.0000 | 5502.0 | 15.24% | 4434.1 | 12.49% | motif file (matrix) | svg |
| 42 | A C G T A G T C A G T C C G A T A C G T A C G T A C T G A C G T A T G C G A C T A C T G T A C G | Sox21(HMG)/ESC-SOX21-ChIP-Seq(GSE110505)/Homer | 1e-51 | -1.179e+02 | 0.0000 | 10598.0 | 29.36% | 9169.4 | 25.83% | motif file (matrix) | svg |
| 43 | C T A G T A C G G A T C G T C A T G C A A C G T T G C A G C T A T C G A T G C A | Hoxa9(Homeobox)/ChickenMSG-Hoxa9.Flag-ChIP-Seq(GSE86088)/Homer | 1e-48 | -1.107e+02 | 0.0000 | 13305.0 | 36.87% | 11786.6 | 33.21% | motif file (matrix) | svg |
| 44 | C T G A A C T G A C T G A G T C A G T C A G C T C T A G T A C G | ZFX(Zf)/mES-Zfx-ChIP-Seq(GSE11431)/Homer | 1e-43 | -9.918e+01 | 0.0000 | 8303.0 | 23.01% | 7113.8 | 20.04% | motif file (matrix) | svg |
| 45 | T G C A A C T G T A C G A T G C A G T C G A C T T C G A A T C G | ZNF711(Zf)/SHSY5Y-ZNF711-ChIP-Seq(GSE20673)/Homer | 1e-42 | -9.679e+01 | 0.0000 | 11742.0 | 32.53% | 10375.3 | 29.23% | motif file (matrix) | svg |
| 46 | T C A G C G T A A G T C A G C T C G T A A G T C C T G A C G T A A G T C G C A T A G T C A G T C A G T C C T G A A C T G T G C A T C G A C A T G A T C G G A T C | Ronin(THAP)/ES-Thap11-ChIP-Seq(GSE51522)/Homer | 1e-38 | -8.857e+01 | 0.0000 | 313.0 | 0.87% | 133.5 | 0.38% | motif file (matrix) | svg |
| 47 | A T G C A G T C G T A C A G C T T C G A C T A G G A T C C T G A G T C A A G T C G C T A T C A G | Rfx5(HTH)/GM12878-Rfx5-ChIP-Seq(GSE31477)/Homer | 1e-38 | -8.775e+01 | 0.0000 | 2469.0 | 6.84% | 1863.1 | 5.25% | motif file (matrix) | svg |
| 48 | G C A T C T A G G T A C A G T C C G A T A C T G C T A G C T A G G T A C G C T A | ZNF416(Zf)/HEK293-ZNF416.GFP-ChIP-Seq(GSE58341)/Homer | 1e-37 | -8.685e+01 | 0.0000 | 9062.0 | 25.11% | 7894.9 | 22.24% | motif file (matrix) | svg |
| 49 | G A C T C A G T A G C T C G A T A G T C G A T C A G T C C G T A A T G C T C A G | Rbpj1(?)/Panc1-Rbpj1-ChIP-Seq(GSE47459)/Homer | 1e-37 | -8.538e+01 | 0.0000 | 8614.0 | 23.87% | 7481.3 | 21.08% | motif file (matrix) | svg |
| 50 | A G C T G C A T A C T G A C G T A G T C A C G T C T A G T A C G | Smad3(MAD)/NPC-Smad3-ChIP-Seq(GSE36673)/Homer | 1e-36 | -8.398e+01 | 0.0000 | 16818.0 | 46.60% | 15362.1 | 43.28% | motif file (matrix) | svg |
| 51 | A G T C A C G T A C T G A G C T A C G T A C G T G T C A A G T C | Foxo1(Forkhead)/RAW-Foxo1-ChIP-Seq(Fan\_et\_al.)/Homer | 1e-36 | -8.323e+01 | 0.0000 | 10186.0 | 28.22% | 8979.0 | 25.30% | motif file (matrix) | svg |
| 52 | G C T A A C G T C T A G G T C A C G T A A C G T C G T A C G A T C A G T A G T C C G T A A C G T C T A G C T G A A T C G | OCT:OCT(POU,Homeobox)/NPC-Brn1-ChIP-Seq(GSE35496)/Homer | 1e-34 | -8.025e+01 | 0.0000 | 80.0 | 0.22% | 13.2 | 0.04% | motif file (matrix) | svg |
| 53 | A G T C C T G A A G T C C G A T C A G T G A T C A T G C A C T G A T C G G A C T | Fli1(ETS)/CD8-FLI-ChIP-Seq(GSE20898)/Homer | 1e-34 | -8.017e+01 | 0.0000 | 7181.0 | 19.90% | 6169.3 | 17.38% | motif file (matrix) | svg |
| 54 | A T G C T C G A A G T C A G C T A C G T G T A C A G T C G C T A C T A G C A T G G T C A C T G A T C A G A G T C | Stat3+il21(Stat)/CD4-Stat3-ChIP-Seq(GSE19198)/Homer | 1e-34 | -7.914e+01 | 0.0000 | 4374.0 | 12.12% | 3589.9 | 10.11% | motif file (matrix) | svg |
| 55 | G A C T A G T C C G A T A C T G C T G A T G A C G T A C C G T A A T C G G C A T C T G A C T A G | Bcl11a(Zf)/HSPC-BCL11A-ChIP-Seq(GSE104676)/Homer | 1e-33 | -7.781e+01 | 0.0000 | 4223.0 | 11.70% | 3458.2 | 9.74% | motif file (matrix) | svg |
| 56 | A T G C G A T C C G T A A G C T C A G T A T C G G C A T A G C T G A C T A C T G | Sox17(HMG)/Endoderm-Sox17-ChIP-Seq(GSE61475)/Homer | 1e-33 | -7.753e+01 | 0.0000 | 3975.0 | 11.01% | 3235.4 | 9.11% | motif file (matrix) | svg |
| 57 | C G T A A C G T A G C T C G A T C T A G G T A C C G T A A G C T C G T A G C T A | Oct4(POU,Homeobox)/mES-Oct4-ChIP-Seq(GSE11431)/Homer | 1e-33 | -7.735e+01 | 0.0000 | 2344.0 | 6.49% | 1787.5 | 5.04% | motif file (matrix) | svg |
| 58 | T C G A A C G T A C G T C T G A G A T C T C A G G A C T G T C A C G T A A G C T G T C A C T A G A G C T A C G T T C G A | NFIL3(bZIP)/HepG2-NFIL3-ChIP-Seq(Encode)/Homer | 1e-33 | -7.734e+01 | 0.0000 | 2771.0 | 7.68% | 2161.6 | 6.09% | motif file (matrix) | svg |
| 59 | C T A G T A G C A G T C G T C A C T G A A C G T C G T A C G T A C G T A G C T A | Hoxd13(Homeobox)/ChickenMSG-Hoxd13.Flag-ChIP-Seq(GSE86088)/Homer | 1e-32 | -7.484e+01 | 0.0000 | 7847.0 | 21.74% | 6823.2 | 19.22% | motif file (matrix) | svg |
| 60 | A G T C G C A T C G T A C G T A G T A C A C G T A C T G G A T C G A T C T C G A | BMYB(HTH)/Hela-BMYB-ChIP-Seq(GSE27030)/Homer | 1e-32 | -7.411e+01 | 0.0000 | 8153.0 | 22.59% | 7115.7 | 20.05% | motif file (matrix) | svg |
| 61 | G C T A A G C T T A C G G T C A C T G A C G A T C T G A G C A T C A G T A G T C | Brn2(POU,Homeobox)/NPC-Brn2-ChIP-Seq(GSE35496)/Homer | 1e-31 | -7.348e+01 | 0.0000 | 435.0 | 1.21% | 230.0 | 0.65% | motif file (matrix) | svg |
| 62 | T A C G C T A G A T G C G A T C G T A C A G T C C T A G A G T C A G T C A G T C G T A C A G T C | Sp1(Zf)/Promoter/Homer | 1e-30 | -7.120e+01 | 0.0000 | 2851.0 | 7.90% | 2254.9 | 6.35% | motif file (matrix) | svg |
| 63 | G T C A A C G T C T A G G T C A C G T A G C A T C G T A C G T A G C A T C A G T A G T C C G T A C A G T C T A G C T G A | OCT:OCT(POU,Homeobox,IR1)/NPC-Brn2-ChIP-Seq(GSE35496)/Homer | 1e-30 | -7.106e+01 | 0.0000 | 117.0 | 0.32% | 31.1 | 0.09% | motif file (matrix) | svg |
| 64 | T A C G T A G C G C T A C G A T C T A G A C G T C A G T C A G T G C T A A G T C G T C A G C A T | FOXK2(Forkhead)/U2OS-FOXK2-ChIP-Seq(E-MTAB-2204)/Homer | 1e-30 | -7.080e+01 | 0.0000 | 3176.0 | 8.80% | 2545.2 | 7.17% | motif file (matrix) | svg |
| 65 | T C G A C T A G A G T C A G T C C G T A C G T A A C G T T A G C T C A G T A C G | NFY(CCAAT)/Promoter/Homer | 1e-29 | -6.885e+01 | 0.0000 | 5172.0 | 14.33% | 4368.3 | 12.31% | motif file (matrix) | svg |
| 66 | T A G C T C A G C A T G G C A T A G C T C G A T A T G C C G T A C G T A G T C A | CHR(?)/Hela-CellCycle-Expression/Homer | 1e-29 | -6.748e+01 | 0.0000 | 3113.0 | 8.63% | 2502.6 | 7.05% | motif file (matrix) | svg |
| 67 | G A C T C T A G C T A G A G T C T G C A A C T G A C G T A C G T C T A G T C A G | AMYB(HTH)/Testes-AMYB-ChIP-Seq(GSE44588)/Homer | 1e-28 | -6.608e+01 | 0.0000 | 8510.0 | 23.58% | 7503.5 | 21.14% | motif file (matrix) | svg |
| 68 | C T G A C T A G T C G A C G T A A T G C C G T A A T C G C G A T T A G C G C A T A T C G G C A T A G C T G A T C G A C T A G C T | ARE(NR)/LNCAP-AR-ChIP-Seq(GSE27824)/Homer | 1e-28 | -6.607e+01 | 0.0000 | 1844.0 | 5.11% | 1389.3 | 3.91% | motif file (matrix) | svg |
| 69 | A G T C G A C T C A G T G T A C A G T C A T C G T C A G A C T G G T C A C G T A | Stat3(Stat)/mES-Stat3-ChIP-Seq(GSE11431)/Homer | 1e-28 | -6.597e+01 | 0.0000 | 3202.0 | 8.87% | 2588.7 | 7.29% | motif file (matrix) | svg |
| 70 | A C T G G T C A A C G T G C A T C G A T T C A G G T A C G T C A A C G T C T G A | Oct11(POU,Homeobox)/NCIH1048-POU2F3-ChIP-seq(GSE115123)/Homer | 1e-28 | -6.497e+01 | 0.0000 | 1646.0 | 4.56% | 1222.2 | 3.44% | motif file (matrix) | svg |
| 71 | C G T A A C G T A C T G G T A C C G T A A C G T C G T A C G T A A C G T A C T G A G T C C G T A A C G T C T G A G C A T | OCT:OCT-short(POU,Homeobox)/NPC-OCT6-ChIP-Seq(GSE43916)/Homer | 1e-27 | -6.432e+01 | 0.0000 | 2710.0 | 7.51% | 2155.5 | 6.07% | motif file (matrix) | svg |
| 72 | T A C G A T C G G A T C G T C A C T G A G C A T C G A T G C T A T C G A G C T A | Unknown(Homeobox)/Limb-p300-ChIP-Seq/Homer | 1e-27 | -6.257e+01 | 0.0000 | 3358.0 | 9.30% | 2743.0 | 7.73% | motif file (matrix) | svg |
| 73 | C T A G T A C G G A C T T G C A T G C A C G A T T A C G C T G A T C G A C T G A | Hoxa10(Homeobox)/ChickenMSG-Hoxa10.Flag-ChIP-Seq(GSE86088)/Homer | 1e-26 | -6.109e+01 | 0.0000 | 2819.0 | 7.81% | 2265.2 | 6.38% | motif file (matrix) | svg |
| 74 | T C G A T A G C G T C A A C T G A C T G C G T A C G T A C T A G A G C T T C A G | ERG(ETS)/VCaP-ERG-ChIP-Seq(GSE14097)/Homer | 1e-25 | -5.928e+01 | 0.0000 | 9709.0 | 26.90% | 8689.8 | 24.48% | motif file (matrix) | svg |
| 75 | T G C A T C G A T C G A A C G T C A T G C G T A A G T C T C A G A C G T G T A C C G T A A G C T | CREB5(bZIP)/LNCaP-CREB5.V5-ChIP-Seq(GSE137775)/Homer | 1e-25 | -5.885e+01 | 0.0000 | 1685.0 | 4.67% | 1274.3 | 3.59% | motif file (matrix) | svg |
| 76 | G C T A C G A T C T A G G T A C G C T A C G A T C G T A G C T A C G A T C A G T G A T C G C T A | Pit1+1bp(Homeobox)/GCrat-Pit1-ChIP-Seq(GSE58009)/Homer | 1e-25 | -5.829e+01 | 0.0000 | 1386.0 | 3.84% | 1019.8 | 2.87% | motif file (matrix) | svg |
| 77 | G A T C G C A T C T G A C G T A A G C T A G C T C G T A T C G A C T G A A C G T G C A T C G T A | Phox2a(Homeobox)/Neuron-Phox2a-ChIP-Seq(GSE31456)/Homer | 1e-24 | -5.599e+01 | 0.0000 | 1648.0 | 4.57% | 1251.1 | 3.52% | motif file (matrix) | svg |
| 78 | T C A G G A C T C A G T C T G A A G C T C T A G G A C T T G C A C T G A A G T C | HLF(bZIP)/HSC-HLF.Flag-ChIP-Seq(GSE69817)/Homer | 1e-24 | -5.552e+01 | 0.0000 | 3525.0 | 9.77% | 2927.4 | 8.25% | motif file (matrix) | svg |
| 79 | G T A C G A C T C G T A C T G A T C G A C G T A G C T A C A G T C T G A T A C G | Mef2a(MADS)/HL1-Mef2a.biotin-ChIP-Seq(GSE21529)/Homer | 1e-23 | -5.522e+01 | 0.0000 | 1861.0 | 5.16% | 1439.4 | 4.06% | motif file (matrix) | svg |
| 80 | T C A G A G C T A T G C C G T A A G T C T C A G A C G T A T C G T C G A A G T C G A T C T G A C | TFE3(bHLH)/MEF-TFE3-ChIP-Seq(GSE75757)/Homer | 1e-23 | -5.481e+01 | 0.0000 | 532.0 | 1.47% | 326.6 | 0.92% | motif file (matrix) | svg |
| 81 | A T G C C T G A A T C G T A C G A G T C C G A T T C A G C G A T C T A G A G C T G T C A G T C A C G T A A G T C C G T A T A C G C T G A | Fox:Ebox(Forkhead,bHLH)/Panc1-Foxa2-ChIP-Seq(GSE47459)/Homer | 1e-23 | -5.401e+01 | 0.0000 | 5278.0 | 14.62% | 4549.4 | 12.82% | motif file (matrix) | svg |
| 82 | C A T G A C G T A G T C G A T C G A T C G A T C G C T A C T A G C T A G C T A G T C A G T C G A | EBF1(EBF)/Near-E2A-ChIP-Seq(GSE21512)/Homer | 1e-20 | -4.813e+01 | 0.0000 | 6478.0 | 17.95% | 5710.9 | 16.09% | motif file (matrix) | svg |
| 83 | C G A T A C G T C G T A C G T A A C G T A G C T T G C A C T G A C G T A A C G T A C G T C G T A | Phox2b(Homeobox)/CLBGA-PHOX2B-ChIP-Seq(GSE90683)/Homer | 1e-20 | -4.746e+01 | 0.0000 | 892.0 | 2.47% | 631.8 | 1.78% | motif file (matrix) | svg |
| 84 | T A G C G T A C C G T A C T A G A C T G T G C A C G T A A T G C C G T A A T C G | AR-halfsite(NR)/LNCaP-AR-ChIP-Seq(GSE27824)/Homer | 1e-20 | -4.695e+01 | 0.0000 | 20634.0 | 57.17% | 19424.0 | 54.72% | motif file (matrix) | svg |
| 85 | T A C G C T G A C A T G G A T C G T A C G C A T T C A G T A C G A G C T G T C A G A T C G C A T T A C G C G T A C T A G G A T C G A T C C G A T A C T G T C A G | ZNF322(Zf)/HEK293-ZNF322.GFP-ChIP-Seq(GSE58341)/Homer | 1e-20 | -4.681e+01 | 0.0000 | 1934.0 | 5.36% | 1534.3 | 4.32% | motif file (matrix) | svg |
| 86 | T C G A T C G A T A G C G T A C T C A G T A C G C G T A C G T A T C A G A G C T | GABPA(ETS)/Jurkat-GABPa-ChIP-Seq(GSE17954)/Homer | 1e-20 | -4.677e+01 | 0.0000 | 5827.0 | 16.15% | 5108.5 | 14.39% | motif file (matrix) | svg |
| 87 | C T A G C T A G A G T C T C A G A C T G A C G T A C G T C T G A | MYB(HTH)/ERMYB-Myb-ChIPSeq(GSE22095)/Homer | 1e-20 | -4.643e+01 | 0.0000 | 9525.0 | 26.39% | 8615.2 | 24.27% | motif file (matrix) | svg |
| 88 | C G A T T A C G G A T C G C T A T C A G G A C T C G A T G T A C G A T C G T C A T C G A T G A C C G T A C T A G G A C T C T A G C T A G G T A C A G T C C G T A | CTCF-SatelliteElement(Zf?)/CD4+-CTCF-ChIP-Seq(Barski\_et\_al.)/Homer | 1e-20 | -4.608e+01 | 0.0000 | 183.0 | 0.51% | 83.9 | 0.24% | motif file (matrix) | svg |
| 89 | G A T C C T G A A G T C C G A T C G A T G A T C A G T C A C T G A T C G A G C T | Elk4(ETS)/Hela-Elk4-ChIP-Seq(GSE31477)/Homer | 1e-19 | -4.496e+01 | 0.0000 | 3892.0 | 10.78% | 3319.0 | 9.35% | motif file (matrix) | svg |
| 90 | T G C A T A G C G A C T T G C A T G A C T G C A C G T A A G C T A G C T A G T C A G T C G T A C | GFY(?)/Promoter/Homer | 1e-19 | -4.391e+01 | 0.0000 | 488.0 | 1.35% | 310.8 | 0.88% | motif file (matrix) | svg |
| 91 | C A T G A G T C A G C T C G T A C G A T C G A T G C A T G C A T C G A T C T G A C A T G T G A C | Mef2d(MADS)/Retina-Mef2d-ChIP-Seq(GSE61391)/Homer | 1e-18 | -4.280e+01 | 0.0000 | 835.0 | 2.31% | 595.4 | 1.68% | motif file (matrix) | svg |
| 92 | T C G A T A G C T G C A A C T G A C T G C G T A C G T A C T A G G A C T T A C G | ETS1(ETS)/Jurkat-ETS1-ChIP-Seq(GSE17954)/Homer | 1e-18 | -4.276e+01 | 0.0000 | 6462.0 | 17.90% | 5734.7 | 16.16% | motif file (matrix) | svg |
| 93 | C T G A A C G T A C G T A C G T A G T C G A C T C G A T C T G A A C T G C G T A C G T A T C G A | STAT5(Stat)/mCD4+-Stat5-ChIP-Seq(GSE12346)/Homer | 1e-18 | -4.245e+01 | 0.0000 | 1956.0 | 5.42% | 1571.7 | 4.43% | motif file (matrix) | svg |
| 94 | C G T A C T G A A C T G A G C T A G T C G A T C G A T C G C A T C T G A C T A G C T A G T A C G T C G A T G C A G C A T | EBF2(EBF)/BrownAdipose-EBF2-ChIP-Seq(GSE97114)/Homer | 1e-18 | -4.203e+01 | 0.0000 | 5456.0 | 15.12% | 4793.5 | 13.50% | motif file (matrix) | svg |
| 95 | A T G C A G T C C T G A A G T C C G A T A C G T A G T C A G T C A C G T A T C G G A C T A C G T | Etv2(ETS)/ES-ER71-ChIP-Seq(GSE59402)/Homer | 1e-18 | -4.179e+01 | 0.0000 | 5712.0 | 15.83% | 5035.2 | 14.19% | motif file (matrix) | svg |
| 96 | T C G A A C G T A C T G C T G A A G T C T C A G A G C T G T A C C G T A A G C T G A T C T C G A | JunD(bZIP)/K562-JunD-ChIP-Seq/Homer | 1e-18 | -4.168e+01 | 0.0000 | 488.0 | 1.35% | 314.7 | 0.89% | motif file (matrix) | svg |
| 97 | T C A G A C G T A G T C T C G A A G T C T C A G G C A T C T A G C T A G A G C T | Usf2(bHLH)/C2C12-Usf2-ChIP-Seq(GSE36030)/Homer | 1e-17 | -4.125e+01 | 0.0000 | 1694.0 | 4.69% | 1343.4 | 3.78% | motif file (matrix) | svg |
| 98 | T C G A C T G A C T G A A T G C G T A C G T C A A G C T A G C T C G T A T C A G | Barx1(Homeobox)/Stomach-Barx1.3xFlag-ChIP-Seq(GSE69483)/Homer | 1e-17 | -4.081e+01 | 0.0000 | 2339.0 | 6.48% | 1922.0 | 5.41% | motif file (matrix) | svg |
| 99 | C A T G A G T C G A C T C G T A C G A T G C A T G A C T G C A T C G A T C T A G C A T G T G A C | Mef2b(MADS)/HEK293-Mef2b.V5-ChIP-Seq(GSE67450)/Homer | 1e-17 | -4.046e+01 | 0.0000 | 3611.0 | 10.01% | 3086.6 | 8.70% | motif file (matrix) | svg |
| 100 | C T G A A G C T A C G T A C G T A G T C G A C T G A C T C T G A C T G A C T A G C G T A C G T A | STAT6(Stat)/CD4-Stat6-ChIP-Seq(GSE22104)/Homer | 1e-17 | -4.044e+01 | 0.0000 | 2790.0 | 7.73% | 2333.9 | 6.58% | motif file (matrix) | svg |
| 101 | C T G A T C A G G T A C G C T A A C T G T G A C G C A T C A T G | SCL(bHLH)/HPC7-Scl-ChIP-Seq(GSE13511)/Homer | 1e-17 | -4.036e+01 | 0.0000 | 23152.0 | 64.15% | 21989.9 | 61.95% | motif file (matrix) | svg |
| 102 | G A C T C A G T G A T C G A T C A C G T G A T C C T G A T A C G C G T A G T C A | STAT6(Stat)/Macrophage-Stat6-ChIP-Seq(GSE38377)/Homer | 1e-17 | -3.954e+01 | 0.0000 | 2878.0 | 7.97% | 2418.1 | 6.81% | motif file (matrix) | svg |
| 103 | A T G C A T G C A T C G T A C G A G C T A G T C G C T A A G T C T C A G G A C T A C T G T C G A | E-box(bHLH)/Promoter/Homer | 1e-16 | -3.911e+01 | 0.0000 | 548.0 | 1.52% | 367.3 | 1.03% | motif file (matrix) | svg |
| 104 | T A C G T C G A G A C T A C T G C T G A A G T C T C A G G A C T T G A C C T G A | Atf1(bZIP)/K562-ATF1-ChIP-Seq(GSE31477)/Homer | 1e-16 | -3.901e+01 | 0.0000 | 3029.0 | 8.39% | 2559.7 | 7.21% | motif file (matrix) | svg |
| 105 | G A C T G T A C T G C A A C G T G A T C G C T A T C G A A C G T A G T C C G T A | Pdx1(Homeobox)/Islet-Pdx1-ChIP-Seq(SRA008281)/Homer | 1e-16 | -3.833e+01 | 0.0000 | 4142.0 | 11.48% | 3591.8 | 10.12% | motif file (matrix) | svg |
| 106 | G T C A G C A T G C T A C A G T C T A G G A T C C G T A C T G A C G T A C G A T | Oct2(POU,Homeobox)/Bcell-Oct2-ChIP-Seq(GSE21512)/Homer | 1e-16 | -3.791e+01 | 0.0000 | 1377.0 | 3.82% | 1076.0 | 3.03% | motif file (matrix) | svg |
| 107 | C G T A A C G T A C G T A C G T A C G T A G T C A G T C C T G A A G C T A G C T | NFAT(RHD)/Jurkat-NFATC1-ChIP-Seq(Jolma\_et\_al.)/Homer | 1e-16 | -3.734e+01 | 0.0000 | 4427.0 | 12.27% | 3863.9 | 10.89% | motif file (matrix) | svg |
| 108 | G A T C T C G A A G T C C G A T C G A T A G T C A T G C A C T G A T C G G A C T | Elk1(ETS)/Hela-Elk1-ChIP-Seq(GSE31477)/Homer | 1e-16 | -3.688e+01 | 0.0000 | 3865.0 | 10.71% | 3343.9 | 9.42% | motif file (matrix) | svg |
| 109 | T A C G A T G C G A C T A C T G A G C T A G T C G T C A T G C A A C G T A G T C G C T A T G C A | Pknox1(Homeobox)/ES-Prep1-ChIP-Seq(GSE63282)/Homer | 1e-15 | -3.576e+01 | 0.0000 | 1402.0 | 3.88% | 1106.4 | 3.12% | motif file (matrix) | svg |
| 110 | C T G A T C A G C A G T C T A G A C T G C T A G G A T C A T C G A C T G C T G A T C A G G A T C | Sp5(Zf)/mES-Sp5.Flag-ChIP-Seq(GSE72989)/Homer | 1e-15 | -3.484e+01 | 0.0000 | 7613.0 | 21.09% | 6891.4 | 19.41% | motif file (matrix) | svg |
| 111 | G T A C G C A T C T G A C G T A G A C T A G C T C A T G T G C A C T G A A C G T G A C T C G T A | Prop1(Homeobox)/GHFT1-PROP1.biotin-ChIP-Seq(GSE77302)/Homer | 1e-14 | -3.377e+01 | 0.0000 | 2588.0 | 7.17% | 2184.9 | 6.16% | motif file (matrix) | svg |
| 112 | C T A G T A C G G A T C G T C A T C G A A C G T T C A G C G T A C G T A C G T A | Hoxd10(Homeobox)/ChickenMSG-Hoxd10.Flag-ChIP-Seq(GSE86088)/Homer | 1e-14 | -3.262e+01 | 0.0000 | 5525.0 | 15.31% | 4929.6 | 13.89% | motif file (matrix) | svg |
| 113 | A T G C G A C T A C G T C T A G A C G T A C G T A C G T C T G A G A T C G C T A A G C T C G T A | Foxa2(Forkhead)/Liver-Foxa2-ChIP-Seq(GSE25694)/Homer | 1e-13 | -3.218e+01 | 0.0000 | 3811.0 | 10.56% | 3324.4 | 9.37% | motif file (matrix) | svg |
| 114 | C T G A A G T C C G A T A G C T A T G C G T A C A C G T A T C G C A G T G C A T | Elf4(ETS)/BMDM-Elf4-ChIP-Seq(GSE88699)/Homer | 1e-13 | -3.210e+01 | 0.0000 | 6297.0 | 17.45% | 5664.1 | 15.96% | motif file (matrix) | svg |
| 115 | T C A G T A C G G A T C G T A C T C G A C G A T C T G A C G T A G C T A C G T A | Hoxd11(Homeobox)/ChickenMSG-Hoxd11.Flag-ChIP-Seq(GSE86088)/Homer | 1e-13 | -3.164e+01 | 0.0000 | 11420.0 | 31.64% | 10580.2 | 29.81% | motif file (matrix) | svg |
| 116 | G A C T T C G A C G A T T A C G C T A G T A C G A C T G A T G C G T A C G T A C | Zac1(Zf)/Neuro2A-Plagl1-ChIP-Seq(GSE75942)/Homer | 1e-13 | -3.143e+01 | 0.0000 | 15612.0 | 43.26% | 14658.6 | 41.30% | motif file (matrix) | svg |
| 117 | G T C A T G C A G C T A A G T C C G T A A C T G T G A C G C A T T C A G C A G T | Ap4(bHLH)/AML-Tfap4-ChIP-Seq(GSE45738)/Homer | 1e-13 | -3.118e+01 | 0.0000 | 7330.0 | 20.31% | 6656.0 | 18.75% | motif file (matrix) | svg |
| 118 | T G C A C T G A A T G C G T C A A C T G A C T G C G T A C G T A C T A G A G C T | Ets1-distal(ETS)/CD4+-PolII-ChIP-Seq(Barski\_et\_al.)/Homer | 1e-13 | -3.093e+01 | 0.0000 | 1791.0 | 4.96% | 1474.2 | 4.15% | motif file (matrix) | svg |
| 119 | T A G C G A T C G T A C C T G A G C A T C T G A C G T A T G C A C G T A G A T C | Hoxa13(Homeobox)/ChickenMSG-Hoxa13.Flag-ChIP-Seq(GSE86088)/Homer | 1e-13 | -3.002e+01 | 0.0000 | 11611.0 | 32.17% | 10783.7 | 30.38% | motif file (matrix) | svg |
| 120 | C A G T C G A T G C A T G C A T G T C A A G C T C A T G C T A G A T G C G T A C | Hoxa11(Homeobox)/ChickenMSG-Hoxa11.Flag-ChIP-Seq(GSE86088)/Homer | 1e-12 | -2.984e+01 | 0.0000 | 10797.0 | 29.92% | 9998.4 | 28.17% | motif file (matrix) | svg |
| 121 | T C A G T C A G G C T A C G T A T A C G G A C T T C A G T C G A C T G A C G T A T A C G G A C T | IRF8(IRF)/BMDM-IRF8-ChIP-Seq(GSE77884)/Homer | 1e-12 | -2.983e+01 | 0.0000 | 1794.0 | 4.97% | 1482.0 | 4.18% | motif file (matrix) | svg |
| 122 | T A G C T A G C G A C T C T A G A G C T A G T C G T C A T G C A A C G T A T G C G C T A T G C A | Pbx3(Homeobox)/GM12878-PBX3-ChIP-Seq(GSE32465)/Homer | 1e-12 | -2.915e+01 | 0.0000 | 1428.0 | 3.96% | 1156.6 | 3.26% | motif file (matrix) | svg |
| 123 | T C G A G C A T A C T G C T G A A G T C T C A G G A C T G T A C C G T A A G C T A G T C G A T C | c-Jun-CRE(bZIP)/K562-cJun-ChIP-Seq(GSE31477)/Homer | 1e-12 | -2.888e+01 | 0.0000 | 1401.0 | 3.88% | 1133.2 | 3.19% | motif file (matrix) | svg |
| 124 | T G C A T C G A T A G C G T A C T C A G C T A G G T C A G C T A T C A G G A C T | ETS(ETS)/Promoter/Homer | 1e-12 | -2.835e+01 | 0.0000 | 2368.0 | 6.56% | 2013.5 | 5.67% | motif file (matrix) | svg |
| 125 | T G C A C T G A A G T C G T C A A C T G A C T G C G T A C G T A C T G A A G C T | EWS:FLI1-fusion(ETS)/SK\_N\_MC-EWS:FLI1-ChIP-Seq(SRA014231)/Homer | 1e-12 | -2.800e+01 | 0.0000 | 3641.0 | 10.09% | 3195.4 | 9.00% | motif file (matrix) | svg |
| 126 | T G C A C T A G C T A G C T G A C A T G A C T G T G C A G A T C G T C A T G C A G T C A G T C A A G C T C T A G G C A T | ZNF675(Zf)/HEK293-ZNF675.GFP-ChIP-Seq(GSE58341)/Homer | 1e-12 | -2.776e+01 | 0.0000 | 748.0 | 2.07% | 563.4 | 1.59% | motif file (matrix) | svg |
| 127 | C T G A T A G C T G A C T C A G C T A G G T C A C G T A T C A G A G C T T C A G | ETV4(ETS)/HepG2-ETV4-ChIP-Seq(ENCODE)/Homer | 1e-11 | -2.751e+01 | 0.0000 | 7213.0 | 19.99% | 6579.4 | 18.54% | motif file (matrix) | svg |
| 128 | A G C T C A T G G C A T G A T C T G C A C T A G G A T C A C G T | Tgif2(Homeobox)/mES-Tgif2-ChIP-Seq(GSE55404)/Homer | 1e-11 | -2.692e+01 | 0.0000 | 15844.0 | 43.90% | 14941.4 | 42.09% | motif file (matrix) | svg |
| 129 | T G A C T A G C T C A G T C G A T C G A C G T A A G T C C G T A C G T A C G A T C T A G T A C G | Sox7(HMG)/ESC-Sox7-ChIP-Seq(GSE133899)/Homer | 1e-11 | -2.653e+01 | 0.0000 | 1660.0 | 4.60% | 1377.3 | 3.88% | motif file (matrix) | svg |
| 130 | C T A G A T G C A T G C C G A T A C T G G A C T A T G C G C T A T G A C A G C T T A G C G C T A | PBX1(Homeobox)/MCF7-PBX1-ChIP-Seq(GSE28007)/Homer | 1e-11 | -2.647e+01 | 0.0000 | 544.0 | 1.51% | 393.4 | 1.11% | motif file (matrix) | svg |
| 131 | T C G A A G T C C G T A A T C G A T G C C G A T A C T G A G T C A G C T A C T G | Tcf12(bHLH)/GM12878-Tcf12-ChIP-Seq(GSE32465)/Homer | 1e-11 | -2.586e+01 | 0.0000 | 5976.0 | 16.56% | 5416.1 | 15.26% | motif file (matrix) | svg |
| 132 | A T G C T C A G T C G A G C A T A C T G C G T A A G T C T C A G G A C T T G A C C G T A A G C T | Atf2(bZIP)/3T3L1-Atf2-ChIP-Seq(GSE56872)/Homer | 1e-11 | -2.554e+01 | 0.0000 | 1523.0 | 4.22% | 1258.8 | 3.55% | motif file (matrix) | svg |
| 133 | C T G A T G C A T A G C T G A C T A C G T C A G C T G A G C T A T C A G G A C T | ELF1(ETS)/Jurkat-ELF1-ChIP-Seq(SRA014231)/Homer | 1e-11 | -2.539e+01 | 0.0000 | 3601.0 | 9.98% | 3177.3 | 8.95% | motif file (matrix) | svg |
| 134 | C T G A A T G C C G T A A C G T A G T C A G T C A C G T A C T G A T C G G C A T | SPDEF(ETS)/VCaP-SPDEF-ChIP-Seq(SRA014231)/Homer | 1e-10 | -2.524e+01 | 0.0000 | 6038.0 | 16.73% | 5481.3 | 15.44% | motif file (matrix) | svg |
| 135 | T A G C G C A T A G T C G A T C A T G C G A C T C T A G A C T G A C T G C T G A A C T G C T A G A G T C T G A C C G A T | GLIS3(Zf)/Thyroid-Glis3.GFP-ChIP-Seq(GSE103297)/Homer | 1e-10 | -2.474e+01 | 0.0000 | 8873.0 | 24.59% | 8201.8 | 23.11% | motif file (matrix) | svg |
| 136 | C G A T C T A G A C G T G T C A C G T A C G T A A G T C C G T A | Foxo3(Forkhead)/U2OS-Foxo3-ChIP-Seq(E-MTAB-2701)/Homer | 1e-10 | -2.417e+01 | 0.0000 | 3403.0 | 9.43% | 3001.9 | 8.46% | motif file (matrix) | svg |
| 137 | C G A T C T A G C T G A A T G C C T G A T C G A C G T A C T G A T C G A T A G C A G T C C G T A A C T G T C G A A T G C | Hand2(bHLH)/Mesoderm-Hand2-ChIP-Seq(GSE61475)/Homer | 1e-10 | -2.408e+01 | 0.0000 | 2719.0 | 7.53% | 2364.4 | 6.66% | motif file (matrix) | svg |
| 138 | T C G A C G T A A G T C A G C T C G T A A G T C T C G A G C T A G A C T C G A T A G T C A G T C A G T C C T G A T C A G T G C A T C G A C A G T A T C G A G T C | GFY-Staf(?,Zf)/Promoter/Homer | 1e-10 | -2.369e+01 | 0.0000 | 428.0 | 1.19% | 304.0 | 0.86% | motif file (matrix) | svg |
| 139 | A T G C G A C T A G C T C T A G C G T A C T A G C G A T C T A G A T C G G A T C | Nkx2.2(Homeobox)/NPC-Nkx2.2-ChIP-Seq(GSE61673)/Homer | 1e-10 | -2.363e+01 | 0.0000 | 10761.0 | 29.82% | 10037.0 | 28.28% | motif file (matrix) | svg |
| 140 | A T G C A T C G T A C G A G C T A T C G C T G A A G T C C T A G A G C T A T G C C T G A A T G C | CRE(bZIP)/Promoter/Homer | 1e-10 | -2.336e+01 | 0.0000 | 1205.0 | 3.34% | 982.8 | 2.77% | motif file (matrix) | svg |
| 141 | C A T G A C T G A G C T A T G C C G T A A T G C G T A C G A C T T A C G C T G A A C T G A C T G G C A T A T G C C T G A | THRb(NR)/HepG2-THRb.Flag-ChIP-Seq(Encode)/Homer | 1e-10 | -2.331e+01 | 0.0000 | 3320.0 | 9.20% | 2930.9 | 8.26% | motif file (matrix) | svg |
| 142 | T C G A A G C T A C G T A C G T A G T C A G T C A C G T A T C G G A C T A T C G | EWS:ERG-fusion(ETS)/CADO\_ES1-EWS:ERG-ChIP-Seq(SRA014231)/Homer | 1e-9 | -2.299e+01 | 0.0000 | 3917.0 | 10.85% | 3493.2 | 9.84% | motif file (matrix) | svg |
| 143 | T C G A C T G A T A G C T G A C T C A G T C A G C G T A C G T A T C A G A G C T | ETV1(ETS)/GIST48-ETV1-ChIP-Seq(GSE22441)/Homer | 1e-9 | -2.295e+01 | 0.0000 | 8445.0 | 23.40% | 7810.1 | 22.00% | motif file (matrix) | svg |
| 144 | T A G C C T A G T C G A G A C T A C T G C T G A A G T C T C A G G C A T T G A C C T G A A G C T | Atf7(bZIP)/3T3L1-Atf7-ChIP-Seq(GSE56872)/Homer | 1e-9 | -2.285e+01 | 0.0000 | 2145.0 | 5.94% | 1842.0 | 5.19% | motif file (matrix) | svg |
| 145 | C T A G C T A G C G T A C G T A T A C G C G A T C T A G C T G A C T G A C G T A T A C G G A C T | PU.1:IRF8(ETS:IRF)/pDC-Irf8-ChIP-Seq(GSE66899)/Homer | 1e-9 | -2.234e+01 | 0.0000 | 1049.0 | 2.91% | 847.9 | 2.39% | motif file (matrix) | svg |
| 146 | C T A G C A T G C A T G T A C G A G T C G C A T A G C T C T A G A C G T A G T C G A C T A C T G A C T G A C T G T C G A | Zfp809(Zf)/ES-Zfp809-ChIP-Seq(GSE70799)/Homer | 1e-9 | -2.213e+01 | 0.0000 | 1418.0 | 3.93% | 1180.6 | 3.33% | motif file (matrix) | svg |
| 147 | T G C A A G C T A C G T C T A G G A T C C T A G G A T C G T C A C T G A A G T C | CEBP(bZIP)/ThioMac-CEBPb-ChIP-Seq(GSE21512)/Homer | 1e-9 | -2.209e+01 | 0.0000 | 2821.0 | 7.82% | 2473.5 | 6.97% | motif file (matrix) | svg |
| 148 | C A T G T A C G T A G C G A T C G A T C A T G C G T A C G A C T T C A G A T G C C G A T A T C G C A G T A C T G G T A C | Zic3(Zf)/mES-Zic3-ChIP-Seq(GSE37889)/Homer | 1e-9 | -2.180e+01 | 0.0000 | 3834.0 | 10.62% | 3425.9 | 9.65% | motif file (matrix) | svg |
| 149 | A G C T A G T C A G T C A C G T C T A G A C G T A C G T A C G T C G T A A G T C G A T C C G T A | FOXP1(Forkhead)/H9-FOXP1-ChIP-Seq(GSE31006)/Homer | 1e-9 | -2.153e+01 | 0.0000 | 2111.0 | 5.85% | 1818.0 | 5.12% | motif file (matrix) | svg |
| 150 | T A G C G T A C A G T C G T A C C G A T A G T C A G T C A G T C A G T C A G T C C G T A G A T C | Zfp281(Zf)/ES-Zfp281-ChIP-Seq(GSE81042)/Homer | 1e-9 | -2.143e+01 | 0.0000 | 1978.0 | 5.48% | 1696.7 | 4.78% | motif file (matrix) | svg |
| 151 | A T C G A G C T A C T G A G T C A C T G A G T C C G T A A C G T A C T G A G T C A C T G A G T C | NRF(NRF)/Promoter/Homer | 1e-9 | -2.118e+01 | 0.0000 | 1289.0 | 3.57% | 1068.1 | 3.01% | motif file (matrix) | svg |
| 152 | A T G C A G C T T C A G T G A C T C A G A T G C T G C A A C G T A T C G G A T C A C T G A G T C | NRF1(NRF)/MCF7-NRF1-ChIP-Seq(Unpublished)/Homer | 1e-9 | -2.109e+01 | 0.0000 | 1191.0 | 3.30% | 980.7 | 2.76% | motif file (matrix) | svg |
| 153 | T G A C T C G A C T G A C T G A A T G C G A T C C T A G T A C G G A C T G A C T G A T C T C G A C T G A C T G A A T G C G A T C C T A G A T C G G A C T G A C T | Tcfcp2l1(CP2)/mES-Tcfcp2l1-ChIP-Seq(GSE11431)/Homer | 1e-9 | -2.105e+01 | 0.0000 | 1008.0 | 2.79% | 816.9 | 2.30% | motif file (matrix) | svg |
| 154 | A T G C A G T C A G C T A G C T A C G T A T C G C G T A C G A T T A G C G A C T | LEF1(HMG)/H1-LEF1-ChIP-Seq(GSE64758)/Homer | 1e-9 | -2.099e+01 | 0.0000 | 3678.0 | 10.19% | 3285.2 | 9.26% | motif file (matrix) | svg |
| 155 | T A C G T C A G A G C T A T G C C G T A A G T C T C A G A C G T A C T G T C G A | USF1(bHLH)/GM12878-Usf1-ChIP-Seq(GSE32465)/Homer | 1e-8 | -2.009e+01 | 0.0000 | 2336.0 | 6.47% | 2036.5 | 5.74% | motif file (matrix) | svg |
| 156 | A C T G A G C T A G T C G T C A A G C T T C A G A T G C G A T C G C A T A T C G T C G A T A G C C G A T C A T G T A G C | Pax8(Paired,Homeobox)/Thyroid-Pax8-ChIP-Seq(GSE26938)/Homer | 1e-8 | -1.978e+01 | 0.0000 | 2091.0 | 5.79% | 1811.2 | 5.10% | motif file (matrix) | svg |
| 157 | C A G T G A C T A G T C G C T A C T G A G T C A C G T A A G C T G T C A C T G A C T G A C T G A A C T G A G C T G A T C | MYNN(Zf)/HEK293-MYNN.eGFP-ChIP-Seq(Encode)/Homer | 1e-8 | -1.965e+01 | 0.0000 | 1212.0 | 3.36% | 1006.2 | 2.83% | motif file (matrix) | svg |
| 158 | T C A G A G C T A T G C C G T A A G C T T C A G C A G T A C T G C T G A A G T C | MITF(bHLH)/MastCells-MITF-ChIP-Seq(GSE48085)/Homer | 1e-8 | -1.957e+01 | 0.0000 | 4641.0 | 12.86% | 4209.5 | 11.86% | motif file (matrix) | svg |
| 159 | C A T G G T A C G A C T G C T A C G T A C G T A C G T A G C T A G A C T C T G A T C A G G T A C | Mef2c(MADS)/GM12878-Mef2c-ChIP-Seq(GSE32465)/Homer | 1e-8 | -1.905e+01 | 0.0000 | 1812.0 | 5.02% | 1558.9 | 4.39% | motif file (matrix) | svg |
| 160 | G A C T G A C T T C G A C G T A C G A T A G C T C T G A A C T G T G A C G A C T T C G A C G T A A C G T A G C T C T G A C T G A G T C A G C T A G C T A C G T A | Pax7(Paired,Homeobox),longest/Myoblast-Pax7-ChIP-Seq(GSE25064)/Homer | 1e-8 | -1.869e+01 | 0.0000 | 131.0 | 0.36% | 75.1 | 0.21% | motif file (matrix) | svg |
| 161 | T G A C C T A G T C A G G T C A C G T A T C A G C G A T T C A G T C G A T G C A C T G A T A G C | PU.1-IRF(ETS:IRF)/Bcell-PU.1-ChIP-Seq(GSE21512)/Homer | 1e-7 | -1.830e+01 | 0.0000 | 7526.0 | 20.85% | 6983.5 | 19.67% | motif file (matrix) | svg |
| 162 | C T A G T C G A C T G A C G T A T A C G G A C T T C A G T C G A G T C A T G C A T A C G A G C T | IRF2(IRF)/Erythroblas-IRF2-ChIP-Seq(GSE36985)/Homer | 1e-7 | -1.826e+01 | 0.0000 | 562.0 | 1.56% | 433.5 | 1.22% | motif file (matrix) | svg |
| 163 | C T A G A T C G G A C T A G T C A G T C A G T C G A C T C T G A A C T G C T A G A C T G C T G A | EBF(EBF)/proBcell-EBF-ChIP-Seq(GSE21978)/Homer | 1e-7 | -1.725e+01 | 0.0000 | 1275.0 | 3.53% | 1076.0 | 3.03% | motif file (matrix) | svg |
| 164 | C G T A C G A T G A C T G A C T T G A C C T G A A T G C C T G A T A G C A G T C A C G T T C G A C A T G T A C G G A C T A T C G G A C T A C G T C T G A T C G A C G T A | Brachyury(T-box)/Mesoendoderm-Brachyury-ChIP-exo(GSE54963)/Homer | 1e-7 | -1.697e+01 | 0.0000 | 1282.0 | 3.55% | 1084.8 | 3.06% | motif file (matrix) | svg |
| 165 | T C G A G A C T A G C T T G A C A G C T G T A C T C A G G A T C A T C G T G C A A C T G C T G A | GFX(?)/Promoter/Homer | 1e-7 | -1.687e+01 | 0.0000 | 125.0 | 0.35% | 73.5 | 0.21% | motif file (matrix) | svg |
| 166 | A G C T T G A C C G A T C G A T C T A G A C G T C A G T C A G T G C T A A G T C | FOXK1(Forkhead)/HEK293-FOXK1-ChIP-Seq(GSE51673)/Homer | 1e-7 | -1.686e+01 | 0.0000 | 4753.0 | 13.17% | 4344.0 | 12.24% | motif file (matrix) | svg |
| 167 | C G T A T A C G T C G A A C T G A C T G C G T A C G T A T A C G A G C T T A C G | PU.1(ETS)/ThioMac-PU.1-ChIP-Seq(GSE21512)/Homer | 1e-7 | -1.663e+01 | 0.0000 | 2877.0 | 7.97% | 2569.6 | 7.24% | motif file (matrix) | svg |
| 168 | G T C A C T G A T C A G G A T C T G C A T G C A A C G T T C A G C G T A C G T A C G T A G C T A | Hoxd12(Homeobox)/ChickenMSG-Hoxd12.Flag-ChIP-Seq(GSE86088)/Homer | 1e-7 | -1.660e+01 | 0.0000 | 8207.0 | 22.74% | 7662.2 | 21.59% | motif file (matrix) | svg |
| 169 | A G T C G A C T A G C T C G A T A T C G G C T A C G A T A T C G C G A T A C T G T A C G A C G T | Tcf7(HMG)/GM12878-TCF7-ChIP-Seq(Encode)/Homer | 1e-7 | -1.646e+01 | 0.0000 | 1814.0 | 5.03% | 1577.9 | 4.45% | motif file (matrix) | svg |
| 170 | A G C T T A C G C G A T T C A G A G C T A C G T G A T C C T G A T A G C A G C T A T G C C T G A C G T A A T C G A G T C C T A G A C T G C T G A G T C A T C G A | PAX6(Paired,Homeobox)/Forebrain-Pax6-ChIP-Seq(GSE66961)/Homer | 1e-7 | -1.615e+01 | 0.0000 | 449.0 | 1.24% | 342.4 | 0.96% | motif file (matrix) | svg |
| 171 | C G A T T G C A T G C A G A T C C G T A A C T G T G A C G A C T C A T G A C T G | Tcf21(bHLH)/ArterySmoothMuscle-Tcf21-ChIP-Seq(GSE61369)/Homer | 1e-6 | -1.602e+01 | 0.0000 | 5689.0 | 15.76% | 5249.0 | 14.79% | motif file (matrix) | svg |
| 172 | A G T C C T A G C T A G A G T C G A T C G T A C A G T C C T A G A G T C A G T C A G T C G T A C | Sp2(Zf)/HEK293-Sp2.eGFP-ChIP-Seq(Encode)/Homer | 1e-6 | -1.550e+01 | 0.0000 | 10076.0 | 27.92% | 9487.1 | 26.73% | motif file (matrix) | svg |
| 173 | T G C A A G C T C A T G C G T A A G C T A C T G G A T C G T C A C G T A A G C T | Atf4(bZIP)/MEF-Atf4-ChIP-Seq(GSE35681)/Homer | 1e-6 | -1.444e+01 | 0.0000 | 1044.0 | 2.89% | 881.6 | 2.48% | motif file (matrix) | svg |
| 174 | A G T C T A G C G A C T A C G T C T A G A C G T A C G T A C G T C T G A A G T C G C T A G A C T C G T A C T A G A C T G | Foxa3(Forkhead)/Liver-Foxa3-ChIP-Seq(GSE77670)/Homer | 1e-6 | -1.441e+01 | 0.0000 | 1425.0 | 3.95% | 1231.6 | 3.47% | motif file (matrix) | svg |
| 175 | C G A T C T A G T C G A A G C T C G A T C T G A C G T A A G C T A C T G C T A G A T G C G A T C | Hoxb4(Homeobox)/ES-Hoxb4-ChIP-Seq(GSE34014)/Homer | 1e-6 | -1.434e+01 | 0.0000 | 853.0 | 2.36% | 708.5 | 2.00% | motif file (matrix) | svg |
| 176 | A T G C C T G A G A C T A C G T A C G T G T A C G A T C C G A T C T A G C A T G C G T A C G T A C T G A G A C T | STAT1(Stat)/HelaS3-STAT1-ChIP-Seq(GSE12782)/Homer | 1e-6 | -1.403e+01 | 0.0000 | 1581.0 | 4.38% | 1378.5 | 3.88% | motif file (matrix) | svg |
| 177 | A T G C G A C T A G C T A G C T A G T C G C T A C A G T C G A T G C T A A C G T A C T G G C T A T A G C G C A T T G A C | IRF:BATF(IRF:bZIP)/pDC-Irf8-ChIP-Seq(GSE66899)/Homer | 1e-6 | -1.386e+01 | 0.0000 | 583.0 | 1.62% | 468.7 | 1.32% | motif file (matrix) | svg |
| 178 | C G T A T A G C T A G C T G C A A C T G C T A G C G T A C G T A T C A G G A C T | EHF(ETS)/LoVo-EHF-ChIP-Seq(GSE49402)/Homer | 1e-5 | -1.370e+01 | 0.0000 | 7370.0 | 20.42% | 6896.0 | 19.43% | motif file (matrix) | svg |
| 179 | A G T C G T A C A G C T C T A G A G T C C G A T A C T G C G T A A C T G G T C A | Zic(Zf)/Cerebellum-ZIC1.2-ChIP-Seq(GSE60731)/Homer | 1e-5 | -1.334e+01 | 0.0000 | 5016.0 | 13.90% | 4637.8 | 13.07% | motif file (matrix) | svg |
| 180 | T G A C G C T A T G A C C G T A T C A G G A T C C G T A C A T G C A T G C T A G C T A G C T A G | Unknown-ESC-element(?)/mES-Nanog-ChIP-Seq(GSE11724)/Homer | 1e-5 | -1.334e+01 | 0.0000 | 3503.0 | 9.71% | 3193.2 | 9.00% | motif file (matrix) | svg |
| 181 | C T G A C T G A T A G C G A T C G C T A G T A C A C G T G A T C T G C A C G T A | Nkx2.5(Homeobox)/HL1-Nkx2.5.biotin-ChIP-Seq(GSE21529)/Homer | 1e-5 | -1.311e+01 | 0.0000 | 11926.0 | 33.04% | 11327.0 | 31.91% | motif file (matrix) | svg |
| 182 | A T G C T A G C A G C T A G C T T G A C G A C T T C A G T A C G G T C A C T G A A T C G T A G C G A C T C A G T A G T C A G C T T C G A A T C G T G C A T G C A | HRE(HSF)/HepG2-HSF1-ChIP-Seq(GSE31477)/Homer | 1e-5 | -1.306e+01 | 0.0000 | 893.0 | 2.47% | 751.9 | 2.12% | motif file (matrix) | svg |
| 183 | G A C T A G C T G T A C G A C T C T G A A C T G G T C A C T G A A T G C T A C G G A C T A C G T A G T C G A C T C T G A | HRE(HSF)/Striatum-HSF1-ChIP-Seq(GSE38000)/Homer | 1e-5 | -1.303e+01 | 0.0000 | 1168.0 | 3.24% | 1003.2 | 2.83% | motif file (matrix) | svg |
| 184 | C T G A C T G A C A T G A T C G A G C T A T C G G A C T C A T G C T G A G T C A | Tbr1(T-box)/Cortex-Tbr1-ChIP-Seq(GSE71384)/Homer | 1e-5 | -1.278e+01 | 0.0000 | 6168.0 | 17.09% | 5751.9 | 16.20% | motif file (matrix) | svg |
| 185 | G C T A A G T C T A C G T G C A A T C G T C A G G C T A T C G A T C A G A G C T | ELF5(ETS)/T47D-ELF5-ChIP-Seq(GSE30407)/Homer | 1e-5 | -1.273e+01 | 0.0000 | 4201.0 | 11.64% | 3865.3 | 10.89% | motif file (matrix) | svg |
| 186 | T G A C C T G A C T A G T C G A C T G A A T G C C G T A A C T G G C A T G T A C G C A T A T C G G C A T A G C T G A T C | PR(NR)/T47D-PR-ChIP-Seq(GSE31130)/Homer | 1e-5 | -1.251e+01 | 0.0000 | 12125.0 | 33.60% | 11531.5 | 32.49% | motif file (matrix) | svg |
| 187 | A C T G G A T C G A C T A C T G A C G T C A T G A C T G A C G T A G C T C G A T | RUNX-AML(Runt)/CD4+-PolII-ChIP-Seq(Barski\_et\_al.)/Homer | 1e-5 | -1.248e+01 | 0.0000 | 3513.0 | 9.73% | 3212.8 | 9.05% | motif file (matrix) | svg |
| 188 | A G T C G A T C G C T A C G A T A C G T T A C G G C A T C T G A G A C T A C T G A G T C G C T A C T G A T C G A C A G T | Oct4:Sox17(POU,Homeobox,HMG)/F9-Sox17-ChIP-Seq(GSE44553)/Homer | 1e-5 | -1.243e+01 | 0.0000 | 594.0 | 1.65% | 484.6 | 1.37% | motif file (matrix) | svg |
| 189 | C T G A T A C G G C A T A G C T A G C T A G T C T C G A A C T G C A G T A G C T A G C T G A T C | IRF3(IRF)/BMDM-Irf3-ChIP-Seq(GSE67343)/Homer | 1e-5 | -1.237e+01 | 0.0000 | 1658.0 | 4.59% | 1462.2 | 4.12% | motif file (matrix) | svg |
| 190 | C G T A C T G A C G T A C T A G T C G A C T A G A C T G C G T A C G T A T A C G A G C T A T C G | SpiB(ETS)/OCILY3-SPIB-ChIP-Seq(GSE56857)/Homer | 1e-5 | -1.223e+01 | 0.0000 | 1329.0 | 3.68% | 1157.5 | 3.26% | motif file (matrix) | svg |
| 191 | T C G A T C G A T A C G G A T C G T C A G T A C C G A T A G C T G T C A T G C A | Nkx3.1(Homeobox)/LNCaP-Nkx3.1-ChIP-Seq(GSE28264)/Homer | 1e-5 | -1.211e+01 | 0.0000 | 12066.0 | 33.43% | 11481.8 | 32.35% | motif file (matrix) | svg |
| 192 | T G A C G T C A A G T C G C T A C T A G A G T C C T G A C A T G A C T G C T A G T A C G T C A G | Zic2(Zf)/ESC-Zic2-ChIP-Seq(SRP197560)/Homer | 1e-5 | -1.206e+01 | 0.0000 | 2945.0 | 8.16% | 2677.8 | 7.54% | motif file (matrix) | svg |
| 193 | T A G C T A G C G C A T C A T G A C T G G C T A C G T A A C G T A C T G G A T C | TEAD4(TEA)/Tropoblast-Tead4-ChIP-Seq(GSE37350)/Homer | 1e-5 | -1.204e+01 | 0.0000 | 4193.0 | 11.62% | 3866.3 | 10.89% | motif file (matrix) | svg |
| 194 | C G T A T G A C T A G C T G C A A C T G A C T G C G T A C G T A T C A G G A C T | ELF3(ETS)/PDAC-ELF3-ChIP-Seq(GSE64557)/Homer | 1e-5 | -1.154e+01 | 0.0000 | 4085.0 | 11.32% | 3769.6 | 10.62% | motif file (matrix) | svg |
| 195 | A G T C G A C T A C T G G A T C G T A C C G T A T G A C A G T C C G A T A G C T A C G T A C G T C T A G G A C T C T G A | ZNF7(Zf)/HepG2-ZNF7.Flag-ChIP-Seq(Encode)/Homer | 1e-4 | -1.144e+01 | 0.0000 | 2667.0 | 7.39% | 2420.3 | 6.82% | motif file (matrix) | svg |
| 196 | A C T G C A T G G C T A T C G A G C T A A G C T A G C T G T A C A G T C T G A C | NFkB-p65-Rel(RHD)/ThioMac-LPS-Expression(GSE23622)/Homer | 1e-4 | -1.119e+01 | 0.0000 | 309.0 | 0.86% | 237.5 | 0.67% | motif file (matrix) | svg |
| 197 | A G C T G A C T C T A G C G T A C A T G C G A T C T A G A T C G G A C T C A G T | Bapx1(Homeobox)/VertebralCol-Bapx1-ChIP-Seq(GSE36672)/Homer | 1e-4 | -1.113e+01 | 0.0000 | 11578.0 | 32.08% | 11024.6 | 31.06% | motif file (matrix) | svg |
| 198 | A T G C A G T C G C T A C G A T C G T A G C A T G C T A C G A T C T A G C A T G T G A C G T C A | CArG(MADS)/PUER-Srf-ChIP-Seq(Sullivan\_et\_al.)/Homer | 1e-4 | -1.094e+01 | 0.0000 | 1437.0 | 3.98% | 1267.4 | 3.57% | motif file (matrix) | svg |
| 199 | G T C A G C A T A C T G G T A C G A C T A C T G G C T A A T C G C A G T G T A C C G T A A G C T | Nrf2(bZIP)/Lymphoblast-Nrf2-ChIP-Seq(GSE37589)/Homer | 1e-4 | -1.087e+01 | 0.0000 | 251.0 | 0.70% | 188.3 | 0.53% | motif file (matrix) | svg |
| 200 | C T A G C A T G C A T G A G C T G A C T C G T A G A T C A G C T G T C A A T G C T C G A C T A G C A T G A C G T A G T C C T G A | LXRE(NR),DR4/RAW-LXRb.biotin-ChIP-Seq(GSE21512)/Homer | 1e-4 | -1.083e+01 | 0.0000 | 238.0 | 0.66% | 177.7 | 0.50% | motif file (matrix) | svg |
| 201 | T C A G C T G A C G T A C G T A T A C G G C A T C T A G C T G A C G T A C G T A T A C G G A C T | IRF1(IRF)/PBMC-IRF1-ChIP-Seq(GSE43036)/Homer | 1e-4 | -1.082e+01 | 0.0000 | 717.0 | 1.99% | 603.5 | 1.70% | motif file (matrix) | svg |
| 202 | T C G A G C A T A T G C C T G A A T G C T A G C A G T C G T A C T C G A A G C T | Srebp1a(bHLH)/HepG2-Srebp1a-ChIP-Seq(GSE31477)/Homer | 1e-4 | -1.068e+01 | 0.0000 | 940.0 | 2.60% | 808.9 | 2.28% | motif file (matrix) | svg |
| 203 | T C G A T C A G T C G A A C T G C A T G A C G T A G T C C T G A | COUP-TFII(NR)/Artia-Nr2f2-ChIP-Seq(GSE46497)/Homer | 1e-4 | -1.066e+01 | 0.0001 | 8683.0 | 24.06% | 8217.8 | 23.15% | motif file (matrix) | svg |
| 204 | G C T A T A G C A G C T A T C G G T C A C G T A G C T A A T G C G A T C C T G A | IRF4(IRF)/GM12878-IRF4-ChIP-Seq(GSE32465)/Homer | 1e-4 | -1.063e+01 | 0.0001 | 2047.0 | 5.67% | 1842.6 | 5.19% | motif file (matrix) | svg |
| 205 | T G C A G C A T C G A T C G T A C A G T A C T G G T A C C G T A C T G A A G C T G T C A A C T G C T A G G T C A C G A T A C T G G T A C T G C A C G T A A G C T | CEBP:CEBP(bZIP)/MEF-Chop-ChIP-Seq(GSE35681)/Homer | 1e-4 | -1.053e+01 | 0.0001 | 604.0 | 1.67% | 502.2 | 1.41% | motif file (matrix) | svg |
| 206 | A T C G A G T C A G T C G A C T A T G C C T G A C T A G A C T G T A C G G T A C C T G A C G A T | AP-2gamma(AP2)/MCF7-TFAP2C-ChIP-Seq(GSE21234)/Homer | 1e-4 | -1.050e+01 | 0.0001 | 6709.0 | 18.59% | 6308.7 | 17.77% | motif file (matrix) | svg |
| 207 | C T A G A T C G G T C A C A T G A G T C G A C T T A C G C A G T A G T C A G T C C T G A C G A T C T A G A T C G G A C T A T C G A G T C G A C T C T A G T C G A | REST-NRSF(Zf)/Jurkat-NRSF-ChIP-Seq/Homer | 1e-4 | -1.040e+01 | 0.0001 | 61.0 | 0.17% | 34.3 | 0.10% | motif file (matrix) | svg |
| 208 | A C T G G A T C C T G A A T C G A G T C T A G C C T G A C G T A T A C G A G T C C T A G C A G T T C A G T C G A T G A C G A T C | PAX5(Paired,Homeobox)/GM12878-PAX5-ChIP-Seq(GSE32465)/Homer | 1e-4 | -1.013e+01 | 0.0001 | 2171.0 | 6.02% | 1964.3 | 5.53% | motif file (matrix) | svg |
| 209 | A C T G C T A G G A T C C G T A C T A G A G T C T G C A G A C T C G T A A C G T T C A G A G T C A C G T C T G A A G T C A G T C G A T C C G T A T C A G T A C G | EBNA1(EBV-virus)/Raji-EBNA1-ChIP-Seq(GSE30709)/Homer | 1e-4 | -9.852e+00 | 0.0001 | 94.0 | 0.26% | 60.8 | 0.17% | motif file (matrix) | svg |
| 210 | C T A G C A T G A C G T A G T C G C T A A G C T A G T C A G C T T C A G C T G A A C T G C A T G G C A T A T G C C G T A | THRa(NR)/C17.2-THRa-ChIP-Seq(GSE38347)/Homer | 1e-4 | -9.777e+00 | 0.0001 | 2239.0 | 6.20% | 2032.1 | 5.72% | motif file (matrix) | svg |
| 211 | T C G A C A T G C A T G A C G T A T G C T C G A C T G A A G C T T A C G T G C A G T A C G A T C A G C T A G T C | FXR(NR),IR1/Liver-FXR-ChIP-Seq(Chong\_et\_al.)/Homer | 1e-4 | -9.769e+00 | 0.0001 | 2019.0 | 5.59% | 1824.6 | 5.14% | motif file (matrix) | svg |
| 212 | T A C G C T G A T C G A C G A T C T A G C T A G T C G A C T G A T C G A T C G A C G T A T C G A G C A T C A T G C G T A T A C G G C A T T G A C C G T A A G C T | NFAT:AP1(RHD,bZIP)/Jurkat-NFATC1-ChIP-Seq(Jolma\_et\_al.)/Homer | 1e-4 | -9.691e+00 | 0.0001 | 757.0 | 2.10% | 646.2 | 1.82% | motif file (matrix) | svg |
| 213 | T A C G T A C G G T A C A T C G T A C G T A C G G T C A C T G A C G T A G A C T | E2F4(E2F)/K562-E2F4-ChIP-Seq(GSE31477)/Homer | 1e-4 | -9.687e+00 | 0.0001 | 2670.0 | 7.40% | 2442.8 | 6.88% | motif file (matrix) | svg |
| 214 | T G C A C T G A C A T G C T A G C A G T A G T C C G T A A T G C A T G C T A C G G C A T T C A G G T C A G A T C G T A C | ERE(NR),IR3/MCF7-ERa-ChIP-Seq(Unpublished)/Homer | 1e-4 | -9.354e+00 | 0.0002 | 1453.0 | 4.03% | 1295.7 | 3.65% | motif file (matrix) | svg |
| 215 | A C T G C A T G T C G A A C G T A C T G C G T A A T C G A C G T G T A C C G T A G A C T A G T C | Fos(bZIP)/TSC-Fos-ChIP-Seq(GSE110950)/Homer | 1e-4 | -9.239e+00 | 0.0002 | 2331.0 | 6.46% | 2126.0 | 5.99% | motif file (matrix) | svg |
| 216 | T A C G T C A G T G C A A G C T T G A C A G C T A G T C A C T G G A T C A C T G T C G A A C T G C T G A C T G A A T G C | ZBTB33(Zf)/GM12878-ZBTB33-ChIP-Seq(GSE32465)/Homer | 1e-3 | -9.077e+00 | 0.0002 | 288.0 | 0.80% | 227.0 | 0.64% | motif file (matrix) | svg |
| 217 | C G A T G A C T C G A T T C A G G A C T A C G T C A G T C T G A G A C T G A C T A G C T C G A T A C T G A T C G G T A C G C T A | NF1:FOXA1(CTF,Forkhead)/LNCAP-FOXA1-ChIP-Seq(GSE27824)/Homer | 1e-3 | -8.970e+00 | 0.0003 | 308.0 | 0.85% | 244.4 | 0.69% | motif file (matrix) | svg |
| 218 | C T G A T C A G A G T C C G T A A T C G A T G C C G A T A C T G A G T C G A C T A T C G A G T C | MyoD(bHLH)/Myotube-MyoD-ChIP-Seq(GSE21614)/Homer | 1e-3 | -8.894e+00 | 0.0003 | 4874.0 | 13.50% | 4564.7 | 12.86% | motif file (matrix) | svg |
| 219 | A T C G T G A C A T G C C T G A T C A G G A C T A G T C C G A T T C A G T C G A C A T G C T A G C T A G C G T A C T A G C T A G C T G A C T A G C T A G A T G C | ZSCAN22(Zf)/HEK293-ZSCAN22.GFP-ChIP-Seq(GSE58341)/Homer | 1e-3 | -8.856e+00 | 0.0003 | 452.0 | 1.25% | 373.4 | 1.05% | motif file (matrix) | svg |
| 220 | C G T A C A T G C A T G A C T G C T A G T C G A G C A T C G A T A G C T A G T C G A T C G T A C | NFkB-p65(RHD)/GM12787-p65-ChIP-Seq(GSE19485)/Homer | 1e-3 | -8.777e+00 | 0.0003 | 2539.0 | 7.03% | 2328.2 | 6.56% | motif file (matrix) | svg |
| 221 | A T G C T C G A T A C G A C G T A T G C A G T C A C G T A G T C A G T C G A T C | Znf263(Zf)/K562-Znf263-ChIP-Seq(GSE31477)/Homer | 1e-3 | -8.603e+00 | 0.0004 | 11007.0 | 30.50% | 10520.5 | 29.64% | motif file (matrix) | svg |
| 222 | G A T C G T A C C G A T A C T G A C T G C G T A C G T A A C G T A C T G G A T C | TEAD(TEA)/Fibroblast-PU.1-ChIP-Seq(Unpublished)/Homer | 1e-3 | -8.466e+00 | 0.0004 | 3105.0 | 8.60% | 2872.6 | 8.09% | motif file (matrix) | svg |
| 223 | T G A C A G T C C T G A T G A C C G T A A C G T A C G T A G T C A G T C C G T A | TEAD1(TEAD)/HepG2-TEAD1-ChIP-Seq(Encode)/Homer | 1e-3 | -7.960e+00 | 0.0007 | 4662.0 | 12.92% | 4375.1 | 12.33% | motif file (matrix) | svg |
| 224 | C G A T C T G A C G T A A G C T A G T C T G C A C T G A A C G T A G C T G C T A | Pax7(Paired,Homeobox)/Myoblast-Pax7-ChIP-Seq(GSE25064)/Homer | 1e-3 | -7.898e+00 | 0.0007 | 387.0 | 1.07% | 319.6 | 0.90% | motif file (matrix) | svg |
| 225 | C T A G A C T G C T A G T C A G T C A G T A C G C T A G A C T G | Maz(Zf)/HepG2-Maz-ChIP-Seq(GSE31477)/Homer | 1e-3 | -7.808e+00 | 0.0008 | 9232.0 | 25.58% | 8808.2 | 24.81% | motif file (matrix) | svg |
| 226 | T A C G T C G A C A G T A C T G G C T A A T G C C G A T G T A C C G T A A C T G T A G C C G T A | NF-E2(bZIP)/K562-NFE2-ChIP-Seq(GSE31477)/Homer | 1e-3 | -7.634e+00 | 0.0009 | 284.0 | 0.79% | 228.1 | 0.64% | motif file (matrix) | svg |
| 227 | C A T G G T A C C G T A A G T C C T A G A C G T A C T G G T A C A G T C A G C T | bHLHE40(bHLH)/HepG2-BHLHE40-ChIP-Seq(GSE31477)/Homer | 1e-3 | -7.456e+00 | 0.0011 | 1480.0 | 4.10% | 1338.3 | 3.77% | motif file (matrix) | svg |
| 228 | A T C G A G C T C T G A C T A G A C T G A C G T G T A C G C T A A T G C A C G T C T A G C A T G T A C G C G A T A T G C C G T A | Reverb(NR),DR2/RAW-Reverba.biotin-ChIP-Seq(GSE45914)/Homer | 1e-3 | -7.294e+00 | 0.0013 | 684.0 | 1.90% | 594.1 | 1.67% | motif file (matrix) | svg |
| 229 | C A T G G A C T T A C G G T C A G T A C G A T C G A C T A G C T A T C G T C G A T A C G T A G C | ERRg(NR)/Kidney-ESRRG-ChIP-Seq(GSE104905)/Homer | 1e-3 | -7.092e+00 | 0.0016 | 4266.0 | 11.82% | 4008.8 | 11.29% | motif file (matrix) | svg |
| 230 | G C A T A T C G C A T G G T A C G C T A A G T C T C A G T G A C G T C A T G C A | Arnt:Ahr(bHLH)/MCF7-Arnt-ChIP-Seq(Lo\_et\_al.)/Homer | 1e-3 | -6.976e+00 | 0.0018 | 3357.0 | 9.30% | 3135.2 | 8.83% | motif file (matrix) | svg |
| 231 | T G C A T G C A A G T C A G T C G A C T C A G T A T G C G A T C C T G A A C G T C T A G C T A G A G T C A C G T A G T C A G T C A G T C G A C T C G T A A C G T A G C T C T A G G A T C G A T C G A T C | ZNF16(Zf)/HEK293-ZNF16.GFP-ChIP-Seq(GSE58341)/Homer | 1e-2 | -6.853e+00 | 0.0020 | 48.0 | 0.13% | 29.6 | 0.08% | motif file (matrix) | svg |
| 232 | A C T G G A C T A G T C C T G A G A T C T C A G A T G C G A C T A G T C A T G C T A G C A G C T A T C G T G C A | PAX5(Paired,Homeobox),condensed/GM12878-PAX5-ChIP-Seq(GSE32465)/Homer | 1e-2 | -6.831e+00 | 0.0020 | 542.0 | 1.50% | 466.1 | 1.31% | motif file (matrix) | svg |
| 233 | C G A T C T G A C G T A C A G T A G T C G A T C G A T C A C T G | Pitx1(Homeobox)/Chicken-Pitx1-ChIP-Seq(GSE38910)/Homer | 1e-2 | -6.693e+00 | 0.0023 | 19729.0 | 54.66% | 19121.1 | 53.87% | motif file (matrix) | svg |
| 234 | G C A T C G T A C G A T G A C T A C T G C T G A G A C T G A T C | Hnf6b(Homeobox)/LNCaP-Hnf6b-ChIP-Seq(GSE106305)/Homer | 1e-2 | -6.598e+00 | 0.0026 | 3461.0 | 9.59% | 3241.9 | 9.13% | motif file (matrix) | svg |
| 235 | A T G C G A T C G A C T A G C T C G A T C G A T G T C A C G A T T C G A A T C G T A G C T A G C | TATA-Box(TBP)/Promoter/Homer | 1e-2 | -6.570e+00 | 0.0026 | 6520.0 | 18.07% | 6199.0 | 17.46% | motif file (matrix) | svg |
| 236 | C T A G T C G A A C G T A C T G C G T A A T G C A C G T G T A C C G T A A G C T G A T C G T A C | Atf3(bZIP)/GBM-ATF3-ChIP-Seq(GSE33912)/Homer | 1e-2 | -6.562e+00 | 0.0026 | 2647.0 | 7.33% | 2460.9 | 6.93% | motif file (matrix) | svg |
| 237 | C T A G G C A T G A T C C G T A A G T C T C A G G A C T C T A G | CLOCK(bHLH)/Liver-Clock-ChIP-Seq(GSE39860)/Homer | 1e-2 | -6.432e+00 | 0.0030 | 2620.0 | 7.26% | 2436.7 | 6.86% | motif file (matrix) | svg |
| 238 | A G C T C T A G T G A C C G T A A C G T C G A T A G T C A G T C C T G A C A T G | TEAD3(TEA)/HepG2-TEAD3-ChIP-Seq(Encode)/Homer | 1e-2 | -6.324e+00 | 0.0033 | 5410.0 | 14.99% | 5128.0 | 14.45% | motif file (matrix) | svg |
| 239 | G T A C G A T C C A G T A G T C A G T C A G T C T G C A G A T C C T G A A T G C G T C A A C G T | WT1(Zf)/Kidney-WT1-ChIP-Seq(GSE90016)/Homer | 1e-2 | -6.279e+00 | 0.0035 | 3908.0 | 10.83% | 3677.2 | 10.36% | motif file (matrix) | svg |
| 240 | A T G C G A T C C G A T C T A G A C T G G C T A C G T A A G C T A C T G A G C T | TEAD2(TEA)/Py2T-Tead2-ChIP-Seq(GSE55709)/Homer | 1e-2 | -6.221e+00 | 0.0036 | 2478.0 | 6.87% | 2303.8 | 6.49% | motif file (matrix) | svg |
| 241 | G C T A A C G T C T A G G T A C G C T A G A C T C T G A G C A T C A T G G A T C | Pit1(Homeobox)/GCrat-Pit1-ChIP-Seq(GSE58009)/Homer | 1e-2 | -6.113e+00 | 0.0040 | 3964.0 | 10.98% | 3734.9 | 10.52% | motif file (matrix) | svg |
| 242 | G A C T T C A G C T A G A G T C A G T C G T A C A G T C C T G A A G T C A G T C A G T C G A C T A G T C A C T G A T G C | KLF3(Zf)/MEF-Klf3-ChIP-Seq(GSE44748)/Homer | 1e-2 | -5.742e+00 | 0.0058 | 3700.0 | 10.25% | 3486.5 | 9.82% | motif file (matrix) | svg |
| 243 | T C A G A C G T T C G A T A G C A G T C C G T A A C T G G T A C A C G T A C T G A T C G A G T C | Atoh1(bHLH)/Cerebellum-Atoh1-ChIP-Seq(GSE22111)/Homer | 1e-2 | -5.686e+00 | 0.0061 | 6236.0 | 17.28% | 5943.2 | 16.74% | motif file (matrix) | svg |
| 244 | G A T C G C A T A C T G C T A G C T G A A G C T G C T A G C T A C T G A T C A G G C A T T G C A A C G T A C G T G A T C G A C T G C A T C T A G T A C G G A C T C T A G C A T G C T A G G T A C T C G A | ZNF136(Zf)/HEK293-ZNF136.GFP-ChIP-Seq(GSE58341)/Homer | 1e-2 | -5.649e+00 | 0.0063 | 384.0 | 1.06% | 328.1 | 0.92% | motif file (matrix) | svg |
| 245 | G C A T C T G A T G C A G A C T A G T C G T A C T C A G C G T A A G C T A C G T G C T A G T A C | Pax7(Paired,Homeobox),long/Myoblast-Pax7-ChIP-Seq(GSE25064)/Homer | 1e-2 | -5.639e+00 | 0.0064 | 93.0 | 0.26% | 68.7 | 0.19% | motif file (matrix) | svg |
| 246 | A C T G C T A G T C G A C G A T C A T G G C T A A T C G C G A T G T A C G C T A A G C T G T A C | Fra1(bZIP)/BT549-Fra1-ChIP-Seq(GSE46166)/Homer | 1e-2 | -5.621e+00 | 0.0065 | 2185.0 | 6.05% | 2032.0 | 5.72% | motif file (matrix) | svg |
| 247 | G A C T C G A T T C A G G A T C G A C T A G C T A G C T A G T C G A T C C G T A C T A G C T A G T C G A T C G A C T G A | Bcl6(Zf)/Liver-Bcl6-ChIP-Seq(GSE31578)/Homer | 1e-2 | -5.579e+00 | 0.0067 | 7636.0 | 21.16% | 7307.7 | 20.59% | motif file (matrix) | svg |
| 248 | T G C A C G T A G T C A A G C T A G T C G C T A T A G C C G A T C T A G G A T C | Gfi1b(Zf)/HPC7-Gfi1b-ChIP-Seq(GSE22178)/Homer | 1e-2 | -5.451e+00 | 0.0076 | 3230.0 | 8.95% | 3038.9 | 8.56% | motif file (matrix) | svg |
| 249 | A G T C G A T C A G T C C G T A A T C G C A G T A G T C G T A C C T G A A C T G T C A G A G C T A G C T A G C T A G C T | PRDM15(Zf)/ESC-Prdm15-ChIP-Seq(GSE73694)/Homer | 1e-2 | -5.343e+00 | 0.0084 | 5957.0 | 16.51% | 5681.0 | 16.00% | motif file (matrix) | svg |
| 250 | A C G T C T A G C G T A A G T C G T A C A C G T A C G T A C G T G T C A G T A C T G A C G A C T | Nur77(NR)/K562-NR4A1-ChIP-Seq(GSE31363)/Homer | 1e-2 | -5.217e+00 | 0.0095 | 757.0 | 2.10% | 678.6 | 1.91% | motif file (matrix) | svg |
| 251 | C G T A C T G A C T A G C T G A A G T C G C T A C G A T A T C G G A C T G A T C A G T C C T G A C T A G C T A G A G T C G C T A C G A T C T A G G A T C G A T C | p73(p53)/Trachea-p73-ChIP-Seq(PRJNA310161)/Homer | 1e-2 | -5.073e+00 | 0.0110 | 238.0 | 0.66% | 198.3 | 0.56% | motif file (matrix) | svg |
| 252 | C T G A A T C G A G C T A G C T A C G T T A G C C T G A T A C G C G A T A C G T G A C T A G T C | ISRE(IRF)/ThioMac-LPS-Expression(GSE23622)/Homer | 1e-2 | -5.014e+00 | 0.0116 | 285.0 | 0.79% | 241.4 | 0.68% | motif file (matrix) | svg |
| 253 | T C A G C T A G C T A G A C T G A C T G G A T C A C T G A C T G C T A G C T A G A G T C G A T C | KLF1(Zf)/HUDEP2-KLF1-CutnRun(GSE136251)/Homer | 1e-2 | -4.955e+00 | 0.0123 | 6292.0 | 17.43% | 6015.8 | 16.95% | motif file (matrix) | svg |
| 254 | G A C T C T A G G A T C C A G T A C T G C T G A A T G C G C A T A T G C C T G A | MafA(bZIP)/Islet-MafA-ChIP-Seq(GSE30298)/Homer | 1e-2 | -4.799e+00 | 0.0143 | 4426.0 | 12.26% | 4207.3 | 11.85% | motif file (matrix) | svg |
| 255 | C G T A C G T A C G T A G C A T G C A T T A C G G T A C G A C T A C T G C G T A A T C G A C G T G T A C C G T A A G C T | Bach1(bZIP)/K562-Bach1-ChIP-Seq(GSE31477)/Homer | 1e-2 | -4.776e+00 | 0.0145 | 254.0 | 0.70% | 214.7 | 0.60% | motif file (matrix) | svg |
| 256 | C T G A A T G C G C T A C G A T A T G C C G T A C G T A C G T A C T A G T A C G | Tcf3(HMG)/mES-Tcf3-ChIP-Seq(GSE11724)/Homer | 1e-2 | -4.646e+00 | 0.0165 | 1394.0 | 3.86% | 1288.9 | 3.63% | motif file (matrix) | svg |
| 257 | C A T G G A C T G C A T A C T G A G C T A C T G A C T G C G T A G C A T A G C T A T C G T A C G | Foxh1(Forkhead)/hESC-FOXH1-ChIP-Seq(GSE29422)/Homer | 1e-2 | -4.643e+00 | 0.0165 | 2660.0 | 7.37% | 2503.7 | 7.05% | motif file (matrix) | svg |
| 258 | C T G A C A T G A C T G A C G T A T G C C G T A C A T G T A C G A T G C G C T A T A C G C T G A C T A G A C T G A C G T A T G C C G T A T A G C | RAR:RXR(NR),DR5/ES-RAR-ChIP-Seq(GSE56893)/Homer | 1e-2 | -4.634e+00 | 0.0166 | 140.0 | 0.39% | 112.7 | 0.32% | motif file (matrix) | svg |
